# The epidemiology of breast cancer in French Guiana 2003–2006

**DOI:** 10.1186/2193-1801-2-471

**Published:** 2013-09-17

**Authors:** Tristan Roue, Angela Fior, Juliette Plenet, Sophie Belliardo, Mathieu Nacher

**Affiliations:** Registre des Cancers de Guyane, Cayenne, French Guiana; Centre d’Investigation Clinique, Epidemiologie Clinique Antilles Guyane, Cayenne, 97300 French Guiana; Centre Hospitalier Andree Rosemon, Cayenne, French Guiana; Laboratoire d’anatomopathologie, Cayenne, 97300 French Guiana

**Keywords:** Breast cancer, French Guiana Registry, Cancer incidence, Cancer in South America

## Abstract

For the first time the incidence and mortality of breast cancer were estimated in French Guiana, an overseas French Territory of South America. A certified cancer registry collected exhaustive data on breast cancer between 2003 and 2005. The age-standardized rate of breast cancer was 47.1 per 100 000 women. The age-standardized death rate was 11.0 per 100 000 women. Although the standardized incidence and death rates were lower than in metropolitan France and South America, the ratio between incidence and mortality showed that the prognosis of breast cancer in French Guiana was worse than in metropolitan France (23 deaths per 100 incident cases versus 17 deaths per 100 incident cases, respectively). The demographics of French Guiana, suggests that mass organized screening may benefit from lowering the age of its target population.

## Introduction

Breast cancer is the most common type of cancer and the most common cause of cancer-related death in women worldwide (Parkin et al. 1999). Breast cancer is also a major public health issue in South America. The incidence is higher in temperate South American countries (69.14 per 100,000 women) than in tropical South American countries (39.07 per 100,000 women) (Schwartsmann 2001). The incidence of breast cancer is higher in developed countries than in less developed countries (Hortobagyi et al. 2005). On the contrary, mortality rates are higher in less developed countries. French Guiana is a French territory located on the Guiana shield. It attracts numerous immigrants from South America and Caribbean who migrate in search of better socioeconomic opportunities. Thus, 30% of the population consists of immigrants (INSEE 2011). The universal healthcare system is that of France, and immigrants have access to health insurance, although in practice this may be somewhat complicated. Cases requiring specialized care are evacuated towards metropolitan France or Martinique for treatment. Since 2005, French Guiana has set up a certified cancer registry. The objective of the present paper is to report, for the first time, the incidence rate of breast cancer and its mortality in this South American territory.

## Methods

The Cancer Registry of French Guiana was created in 2005 and is housed by the Regional Union of health care professionals (URPS). It has been officially certified by the Comité National des Registres (CNR) an emanation of the INSERM (National Institute for Medical Research) and the INVS (National Institute for Epidemiologic Surveillance and Alert, French CDC) in 2010. Every three years the registry undergoes a quality audit by the Comité National des Registres. The objective of the registry is to compile all patients living in French Guiana with malignant invasive pathology and/or in situ lesions starting January 1st 2003 in persons living in French Guiana, whatever the tumoral location and the place of diagnosis and care. Benign tumours, recurrences and metastases, and cancers of patients residing outside of French Guiana are excluded. Cutaneous basocellular carcinoma and pituitary gland tumours are excluded.

The registry is staffed by 3 trained research technicians, a statistician, a secretary and two medical doctors. It has a scientific board and an ethical review board. The database has national approval by the Comission Nationale Informatique et Libertés (CNIL). All confidential information, within the Registry is encoded, protected by security systems and destroyed when no longer needed. No published results can enable the identification of individuals (Roue et al. 2012).

Cancer cases must be confirmed on at least 2 sources. Between 2003 and 2006 one breast cancer case was not included since it was not confirmed by a second source. The search for the cases is active. The three main sources are pathologists, medical information offices of public and private hospitals and health insurance notifications. After first notification of cases, every medical file is checked in different laboratories, private and public hospital departments, administrative services and medical specialists in French Guiana and outside the registration area. Arrangements have been made with the hospitals outside the registration area to notify the registry of any resident cancer cases that they diagnose and treat. The capture-recapture method was used to estimate the completeness of cancer registration. The principle of the capture-recapture method is that by comparing cases in three overlapping data sources, the number of unascertained cases can be estimated. The validity of the estimate relies on 3 basic assumptions: cases must be accurately matched, study population is closed and the individuals have equal catchability. To take into account all possible two-way interactions (dependencies) between the data sources and correct for these, log-linear models were fitted to the observed cases producing a maximum likelihood estimate for unascertained cases. The model fit can be evaluated by comparing p-values and/or the Akaikes Information Criterion (AIC) (an der Heiden 2009). The model with the two interaction terms between pathologists and medical information offices of public and private hospitals and between pathologists and health insurance notifications, was selected as the best fitting (AIC = −1.93) model. According to this model, 4 cases of breast cancer were missed between 2003 and 2006: completeness was estimated to be 97%. Retrospective data regarding the patient identity, demographics, diagnosis, date of diagnosis, stage of disease, the nature, the place and the date of treatment are recorded. The vital status of patients is checked regularly. The follow-up for the vital status is important to evaluate survival after diagnosis and treatment, and it can be done actively through the RNIPP (Repertoire National d’Identification des Personnes Physiques) an emanation of the Insee (Institut national de la statistique et des études économiques), or passively by matching death certificates in the town council. Because patients often need medical transfer to metropolitan France or Martinique, 208 sources were consulted to collect the required data for breast cancer and the average number of sources per registered case is over six. Duplicate observations are removed. Data coding strictly follows procedures of the FRANCIM (Réseau Français des Registres de Cancer) & ENCR (European Network of Cancer Registries) network. Tumour topography and morphology are coded according to the international classification of diseases for oncology (ICM-O-3). The TNM stage is coded according to the International Union against Cancer (edition of the studied years).

Data is entered on MOViBASE and was analysed using STATA 11®. The total number of cancer cases was reported, a crude incidence rate was calculated with population of French Guiana as a reference. Comparability of incidence rates is ensured by using age-standardized rates using the world population as a reference (direct standardization). Mortality data was obtained through the INSERM database (CépiDC) which compiles data from death certificates throughout France.

## Results

**Figure 1:**
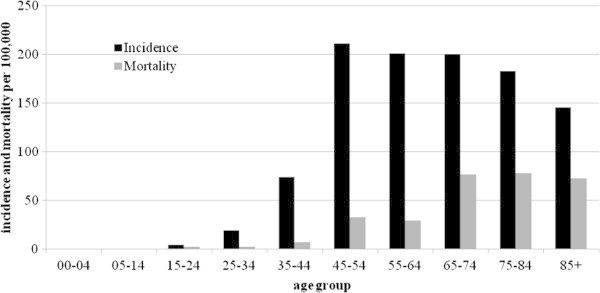
**Incidence and mortality per 100,000 women by age group in French Guiana between 2003 and 2006.**

The median age at diagnosis was 50 years (interquartile range: 44–59 years), the youngest patient was 27 years old and the oldest was 92 years old. Between 2003 and 2006, there were 147 new cases of breast cancer, which corresponded to a crude incidence rate of 36.7 per 100,000 women (95% confidence interval [CI], 30.8–42.6). The standardized incidence rate was 47.1 per 100,000 women (95% CI, 39.0–55.2). Overall breast cancer represents 27% of all cancers in women. The incidence of breast cancer grew significantly after 40 years of age and peaked at 211.2 per 100,000 women between 45 and 54 years and then decreases after 55 years of age (Figure 1).

The median age at death from breast cancer was 52 years (interquartile range: 46–64 years), the youngest patient was 28 years old and the oldest was 85 years old. In French Guiana, breast cancer is the first case of cancer related death in women and the 4^th^ cause of cancer-related death in the global population. The crude mortality rate by breast cancer was 8.0 per 100,000 women (95% CI, 5.2–10.8). The standardized mortality rate was 11.0 deaths per 100,000 women (95% CI, 7.0–15.1).

Histoprognostic grading according to Scarff Bloom and Richardson’s classification (SBR) showed that 12% were SBRI (good prognosis), 44% were SBRII (intermediate prognosis), and 34% were SBRIII (unfavourable prognosis). For 10% the data was unavailable.

The staging was assigned to 1 of 5 categories: the staging at the time of diagnosis showed that 20% of T1N0M0 (tumour ≤ 2 cm, node-negative), 20% of T2–3N0M0 (tumour ≥ 2 cm and ≤ 5 cm, node-negative), 39% of T1–3 N + M0 (tumour > 5 cm, node-positive, regardless of the number and anatomic level of the involved nodes in the axilla), 5% of T4N×M0 (any size with extension to the skin or the thorax wall, regardless of nodal status), 9% of M1 (metastatic and unspecified stage). Between 2003 and 2006, 8% of breast cancer cases were in situ lesions.

Over 96% of women benefited from treatment within 8 weeks, among which 64% outside of French Guiana. Within 4 months following the diagnosis, surgery concerned 76% of treated patients (among whom 73% outside of French Guiana), chemotherapy 54% (among whom 78% outside of French Guiana), radiotherapy for 8% (always outside of French Guiana), and hormone therapy for 9% (among whom 70% outside of French Guiana). Eighty four percent of surgical interventions were considered to be curative, the limits of the surgery were free of any remaining tumour.

## Discussion

**Figure 2:**
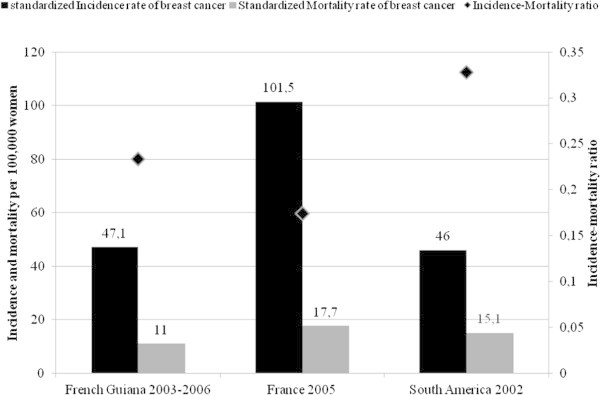
**Incidence rate and mortality rate of breast cancer.**

**Figure 3:**
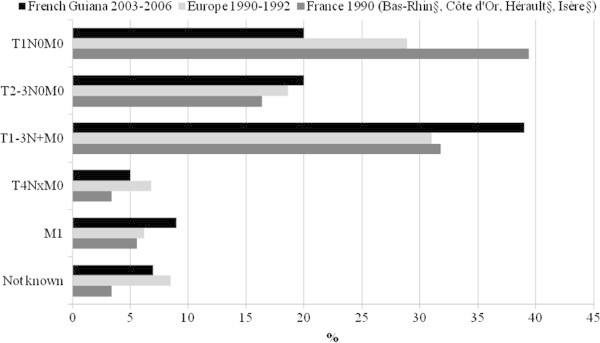
**Comparison of stage at diagnosis of breast cancer between French Guiana, Europe and four French cancer registries.**

In French Guiana, the incidence of breast cancer is on par with the overall incidence in South America, but slightly higher than the incidence in tropical South America (Schwartsmann 2001). In contrast the standardized incidence of breast cancer in this French overseas territory is markedly lower than in metropolitan France (Belot et al. 2009). The standardized death rate was lower in French Guiana than in South America and metropolitan France (Belot et al. 2009). However, the ratio between mortality and incidence of breast cancer may indirectly reflect the influence of early diagnosis. Here the ratio is higher than in metropolitan France, but lower than South America (Figure 2). Thus the prognosis of breast cancer in French Guiana is worse than in France with 23 deaths per 100 incident cases against 17 per 100 in metropolitan France. This excess may be explained by a later diagnosis. At the time of diagnosis 39% of women had T1–3 N + M0 (tumour > 5 cm, node-positive) and 9% had a metastatic stage. Figure 3 shows that, in this ultraperipheric region of Europe, there is a statistically significant diagnostic delay compared to earlier data from mainland Europe and France (Sant et al. 2003).

Since 2004, mass organized screening was initiated throughout the French territory. All women aged 50–74 years are concerned by this every 2 years. In French Guiana, this strategy was initiated in 2005 by the Guianese Association for Organized Cancer Screening (AGDOC). Because of the young age of the population in French Guiana, the median age of women at the time of diagnosis is 50 years against 61 in metropolitan France. Fifty percent of breast cancer cases are diagnosed among women aged <50 years old whereas this figure is 21% in metropolitan France. This suggests that lowering the age of women targeted by organized screening may be warranted given the local epidemiology.
